# Supplementary material for: Polyketide synthase gene clusters in the ericoid mycorrhizal fungus Oidiodendron maius: genome-wide prediction, expression profiling, and functional characterization of knockout mutants
Source: Front Plant Sci. 2026 Sep 16;17:1924430. doi: 10.3389/fpls.2026.1924430 (PMC13623903; doi:10.3389/fpls.2026.1924430)
Supplement: Supplementary file 1 [file SupplementaryFile1.zip › Supplementary Materials/Supplementary Folder 1.zip!/Oidma1_AssemblyScaffolds_Repeatmasked/knownclusterblast/cluster1/jgi.p_Oidma1_36616_mibig_hits.html]

| MIBiG Protein | Description | MIBiG Cluster | MiBiG Product | % ID | % Coverage | BLAST Score | E-value |
| --- | --- | --- | --- | --- | --- | --- | --- |
| EED53480.1 | O-methyltransferase,\_putative | BGC0001304 | Aflavarin\_biosynthetic\_gene\_cluster | 28 | 98.5148514851 | 148 | 1.8e-35 |
| EAL85127.1 | O-methyltransferase,\_putative | BGC0001067 | Fumagillin\_biosynthetic\_gene\_cluster | 36 | 61.3861386139 | 144 | 2.5e-34 |
| AAA67518.1 |  | BGC0000275 | Tetracenomycin\_biosynthetic\_gene\_cluster | 27 | 82.4257425743 | 92 | 8.7e-19 |
| AKD43502.1 | O-Methyltransferase | BGC0001409 | Dutomycin\_biosynthetic\_gene\_cluster | 24 | 92.3267326733 | 81 | 2e-15 |
| ACN64847.1 | PokMT3 | BGC0001061 | Polyketomycin\_biosynthetic\_gene\_cluster | 25 | 97.2772277228 | 81 | 3.4e-15 |
| AFD30960.1 | CrmM | BGC0000966 | Caerulomycin\_A\_biosynthetic\_gene\_cluster | 25 | 86.8811881188 | 78 | 2.2e-14 |
| ACB47081.1 | DynO6 | BGC0001060 | Dynemicin\_biosynthetic\_gene\_cluster | 31 | 43.0693069307 | 74 | 2.5e-13 |
| sln4 | methyltransferase | BGC0001230 | Salinamides\_biosynthetic\_gene\_cluster | 25 | 84.1584158416 | 67 | 3.9e-11 |
| AFV52157.1 | O-methyltransferase | BGC0000081 | Kedarcidin\_biosynthetic\_gene\_cluster | 26 | 83.6633663366 | 66 | 8.8e-11 |
| AAP69580.1 | putative\_O-methyltransferase | BGC0000226 | Gilvocarcin\_biosynthetic\_gene\_cluster | 25 | 50.9900990099 | 64 | 4.3e-10 |
| BAB69170.1 | O-methyltransferase | BGC0000271 | Spore\_pigment\_biosynthetic\_gene\_cluster | 29 | 38.1188118812 | 60 | 6.3e-09 |
| AEZ64592.1 | O-methyltransferase | BGC0001065 | Herboxidiene\_biosynthetic\_gene\_cluster | 25 | 82.6732673267 | 54 | 3.4e-07 |
| AHZ61883.1 | Lom49 | BGC0000240 | Lomaiviticin\_biosynthetic\_gene\_cluster | 30 | 37.1287128713 | 53 | 5.9e-07 |
| ABP54665.1 | O-methyltransferase,\_family\_2 | BGC0000241 | Lomaiviticin\_biosynthetic\_gene\_cluster | 30 | 36.8811881188 | 50 | 3.8e-06 |
